# Neuromodulatory Effects of Arecoline on Anxiety-like Behavior in Mice Exposed to Chronic Unpredictable Mild Stress

**DOI:** 10.3390/ijms27010371

**Published:** 2025-12-29

**Authors:** Xiangfei Zhang, Danyang Wang, Jingwen Cui, Bei Fan, Fengzhong Wang, Cong Lu

**Affiliations:** 1 Institute of Food Science and Technology, Chinese Academy of Agricultural Sciences, Beijing 100193, China; 82101235017@caas.cn (X.Z.); w15873392907@126.com (D.W.); 18734021485@163.com (J.C.); fanbei517@163.com (B.F.); 2 College of Food Science and Engineering, Shanxi Agricultural University, Jinzhong 030801, China; 3 Institute of Food and Nutrition Development, Ministry of Agriculture and Rural Affairs, Beijing 100081, China

**Keywords:** arecoline, *Areca catechu*, chronic unpredictable mild stress, anxiety, hippocampus, oxidative stress, neuroplasticity

## Abstract

Chronic stress disrupts neuroendocrine regulation, neurotransmitter balance, and neuronal redox homeostasis, thereby contributing to the development of anxiety-related neuropathology. Arecoline, the predominant alkaloid of *Areca catechu* L., displays diverse neuropharmacological properties, yet its role in stress-induced emotional dysfunction has not been fully elucidated. This study examined the anxiolytic-like and neuroprotective effects of arecoline in mice exposed to chronic unpredictable mild stress (CUMS). Arecoline administration markedly improved behavioral outcomes, reflected by increased central exploration in the open-field test, prolonged time in the light compartment, and enhanced open-arm activity in the elevated plus maze. These behavioral benefits were accompanied by normalization of serum corticosterone levels, restoration of hippocampal neurotransmitters, reinforcement of antioxidant enzyme activities, and attenuation of pro-inflammatory cytokines. At the molecular level, arecoline elevated brain-derived neurotrophic factor (BDNF), tropomyosin receptor kinase B (TrkB), cAMP response element-binding protein (CREB), N-methyl-D-aspartate receptor (NMDAR), and Ca^2+^/calmodulin-dependent protein kinase II (CaMKII), indicating enhanced synaptic plasticity, while concurrently diminishing oxidative and inflammatory stress. Collectively, the findings suggest that arecoline exerts multifaceted neuroprotective actions under chronic stress by coordinating neuroendocrine modulation, neurotransmitter homeostasis, antioxidant defenses, and synaptic plasticity. This study provides new mechanistic evidence supporting the potential relevance of arecoline as a functional neuroactive compound for managing stress-induced anxiety disorders.

## 1. Introduction

Anxiety disorders rank among the most widespread mental health conditions globally, impacting roughly a third of the population at some point in their lives and placing a heavy burden on public health systems [1,2]. Besides psychological symptoms, anxiety is frequently comorbid with cardiovascular, metabolic, and neurodegenerative diseases, further exacerbating overall morbidity. Current pharmacological therapies, including benzodiazepines and selective serotonin reuptake inhibitors (SSRIs), offer only partial efficacy and are often accompanied by sedation, dependence, and withdrawal complications [3,4]. These limitations underscore the need for safer and more effective anxiolytic agents.

Chronic stress is recognized as a major trigger of anxiety, initiating a cascade of neuroendocrine, neurochemical, and cellular disturbances. Amplified activity in the hypothalamic–pituitary–adrenal (HPA) axis boosts corticosterone (CORT) levels and disrupts feedback regulation in stress- prone regions, leading to maladaptive emotional responses [5]. This dysregulation alters neurotransmitter balance, particularly in serotonergic, dopaminergic, noradrenergic, and γ-aminobutyric acid (GABA) systems, resulting in emotional instability and cognitive dysfunction [6]. In parallel, oxidative stress and neuroinflammation arise as key downstream events. Reactive oxygen species (ROS) overproduction and lipid peroxidation compromise neuronal integrity, while proinflammatory cytokines amplify neuronal injury [7,8]. These redox–immune interactions form a self-perpetuating cycle that accelerates neurodegeneration. Prolonged stress further impairs neuroplasticity-related pathways, including brain-derived neurotrophic factor (BDNF)/tropomyosin receptor kinase B (TrkB)/cAMP response element-binding protein (CREB) and N-methyl-D-aspartate receptor (NMDAR)/Ca^2+^/calmodulin-dependent protein kinase II (CaMKII) signaling, leading to synaptic dysfunction and persistent anxiety [9]. Collectively, these alterations indicate that anxiety is a multifactorial disorder involving neuroendocrine imbalance, neurotransmitter dysregulation, oxidative damage, and reduced neuroplasticity.

Given this complexity, natural products have emerged as promising alternatives or adjuncts to conventional therapy [10]. Plant-derived polyphenols and alkaloids exhibit neuroprotective, redox-regulating, and inflammation-modulating effects [11]. Experimental studies indicate that flavonoids, isoflavones, and plant-derived polysaccharides can relieve anxiety-like behaviors in rodents by rebalancing neurotransmitter signaling and reducing oxidative and inflammatory disturbances [12,13,14]. Such multi-target actions may confer advantages over single-mechanism synthetic drugs, providing efficacy with improved safety profiles [15].

*Areca catechu* L. (Arecaceae) is a traditional medicinal and psychoactive plant used in Chinese, Ayurvedic, and Southeast Asian medicine to regulate mood and circulation [16]. Its major alkaloid, arecoline, exhibits antioxidant [17,18], anti-inflammatory [19], neuroprotective [20,21], and neuromodulatory activities [22,23]. Preclinical evidence indicates that arecoline can modulate central neurotransmission [24], regulate cholinergic signaling and synaptic transmission [25], and alleviate inflammation-induced neuronal injury by activating the antioxidant pathway [18]. Despite these pharmacological activities, its potential as an anxiolytic agent has not been systematically investigated. In particular, whether arecoline can alleviate chronic unpredictable mild stress (CUMS)-induced anxiety-like behaviors and through which molecular mechanisms remains largely unknown.

Therefore, this study aimed to evaluate the anxiolytic efficacy of arecoline in mice subjected to CUMS. Behavioral outcomes were assessed using the open field (OFT), light–dark (LDB), and elevated plus maze (EPM) tests, while biochemical and molecular analyses examined oxidative stress markers, inflammatory cytokines, neurotransmitters, and neuroplasticity-related proteins. Particular focus was given to the BDNF–TrkB–CREB and NMDA receptor–CaMKII pathways and HPA axis regulation. This work provides mechanistic insight into the anxiolytic potential of arecoline and supports the pharmacological basis of A. catechu in stress-related disorders.

## 2. Results

### 2.1. Effects of Arecoline on Exploratory Behavior in Mice Subjected to CUMS in the Open Field Test

**Figure 1:**
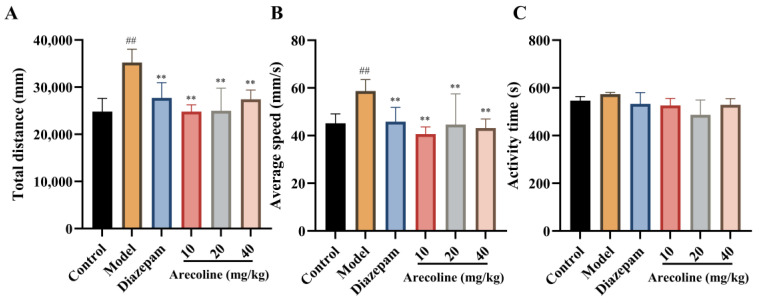
Effects of arecoline on exploratory movement in the OFT. (**A**) Total distance; (**B**) Average speed; (**C**) Activity time. Values represent the mean ± SEM (*n* = 12). ## *p* < 0.01 vs. control; ** *p* < 0.01 vs. model.

As shown in Figure 1A–C, exposure to CUMS caused a remarkable elevation in both total distance traveled and average movement speed compared with the control group (## *p* < 0.01), reflecting stress-induced hyperlocomotion and heightened arousal. Administration of diazepam effectively counteracted these effects and restored locomotor parameters to near-baseline levels, consistent with its recognized anxiolytic activity. Arecoline administration (10, 20, 40 mg/kg) markedly reduced total distance and average velocity versus the CUMS model group (** *p* < 0.01), Which indicates that arecoline attenuates stress-evoked hyperactivity under CUMS conditions. In contrast, total activity time showed no statistical difference among groups, suggesting that the observed effects were not attributable to sedation but reflected modulation of stress-related motor excitation. It should be noted that all arecoline treatments were administered exclusively to CUMS-exposed mice.

### 2.2. Effects of Arecoline on Anxiety-Related Behaviors in Mice Subjected to CUMS in the Light–Dark Box Test

**Figure 2:**
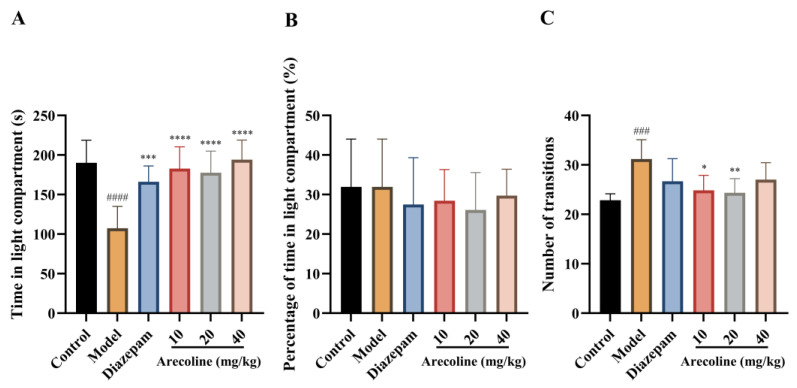
Effects of arecoline on anxiety-like behavior in the LDB test of CUMS-induced mice. (**A**) Time in light compartment; (**B**) Percentage of time in light compartment; (**C**) Number of transitions between light and dark compartments. Values represent the mean ± SEM (*n* = 12). ### *p* < 0.001, #### *p* < 0.0001 vs. control; * *p* <0.05, ** *p* < 0.01, *** *p* < 0.001, **** *p* < 0.0001 vs. model.

As illustrated in Figure 2A–C, mice subjected to CUMS showed a marked decrease in the time spent in the light compartment (#### *p* < 0.0001) and exhibited an increased number of transitions between the light and dark compartments (### *p* < 0.001), indicating stress-induced behavioral alterations in the LDB test. Administration of diazepam increased the time spent in the light compartment (*** *p* < 0.001). Treatment with arecoline (10, 20, and 40 mg/kg) markedly increased the time mice remained in the illuminated compartment (**** *p* < 0.0001), showing a robust anxiolytic-like effect comparable to diazepam in this readout. In contrast, the number of transitions was moderately reduced relative to the CUMS model group at 10 and 20 mg/kg (* *p* < 0.05, ** *p* < 0.01), while no significant change was observed at 40 mg/kg.

### 2.3. Effects of Arecoline on Elevated Plus Maze Performance in Mice Subjected to CUMS

**Figure 3:**
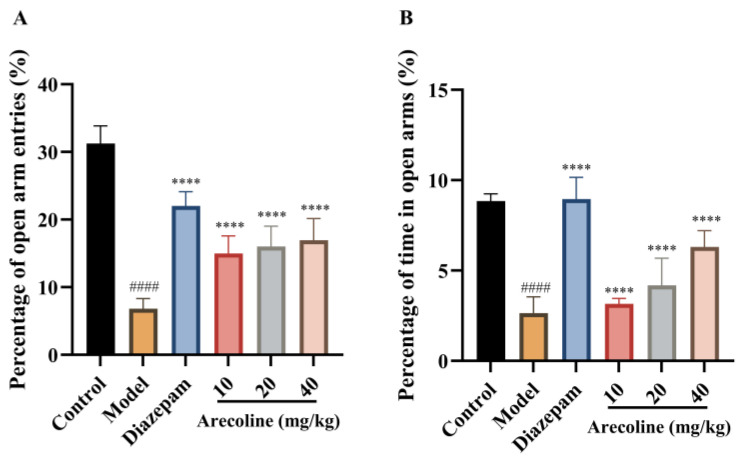
Effects of arecoline on EPM-related behavioral performance in CUMS-induced mice. (**A**) Percentage of open arm entries; (**B**) Percentage of time spent in open arms. Values represent the mean ± SEM (*n* = 12). #### *p* < 0.0001 vs. control; **** *p* < 0.0001 vs. model.

As shown in Figure 3A,B, mice subjected to CUMS exhibited a pronounced reduction in both the percentage of open-arm entries and the percentage of time spent in the open arms compared with the control group (#### *p* < 0.0001), indicating a robust anxiety-like behavioral phenotype in the elevated plus maze. Diazepam treatment significantly increased the percentage of open-arm entries and the percentage of time spent in the open arms relative to the CUMS model group (**** *p* < 0.0001), validating the sensitivity of the EPM paradigm to anxiolytic intervention. Similarly, arecoline administration at doses of 10, 20, and 40 mg/kg significantly elevated both open-arm entry percentage and open-arm time percentage compared with the model group (**** *p* < 0.0001). Although all arecoline-treated groups showed clear improvements relative to the CUMS group, no strict dose-dependent trend was observed across the tested dose range. Collectively, these results demonstrate that arecoline effectively attenuates CUMS-induced reductions in open-arm exploration in the elevated plus maze, consistent with an anxiolytic-like effect.

### 2.4. Effects of Arecoline on Serum Corticosterone Levels in Mice Subjected to CUMS

**Figure 4:**
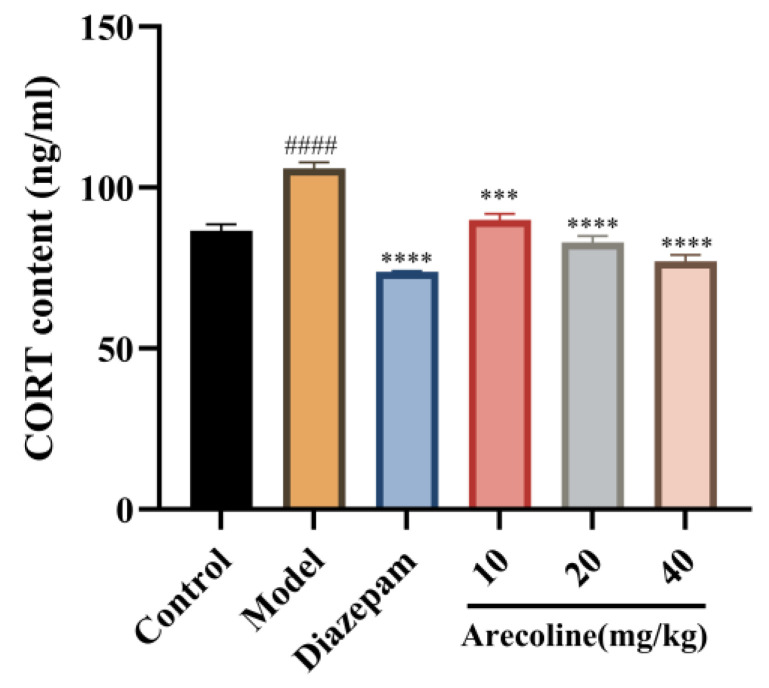
Effects of arecoline on serum CORT levels in CUMS-induced mice. Values represent the mean ± SEM (*n* = 12). #### *p* < 0.0001 vs. control; *** *p* < 0.001, **** *p* < 0.0001 vs. model.

As illustrated in Figure 4, CUMS markedly increased CORT content in the serum compared with the control group (#### *p* < 0.0001), indicating sustained hyperactivation of the HPA axis and excessive stress hormone release into the circulation. Treatment with diazepam significantly reduced serum CORT levels (**** *p* < 0.0001), validating the model’s responsiveness to anxiolytic intervention. Similarly, Arecoline administration (10, 20, 40 mg/kg) markedly reduced serum CORT levels compared with the CUMS group (*** *p* < 0.001, **** *p* < 0.0001). Among these, the 20 mg/kg and 40 mg/kg doses nearly normalized serum CORT levels to control values, suggesting that arecoline mitigates chronic stress–induced endocrine overactivation and contributes to restoring systemic homeostasis of the HPA axis. These results demonstrate that arecoline attenuates CUMS-induced HPA axis hyperactivity by downregulating circulating corticosterone levels, consistent with its observed anxiolytic-like behavioral effects.

### 2.5. Effects of Arecoline on Monoamine Neurotransmitter and GABA Levels in the Hippocampus of Mice Subjected to CUMS

**Figure 5:**
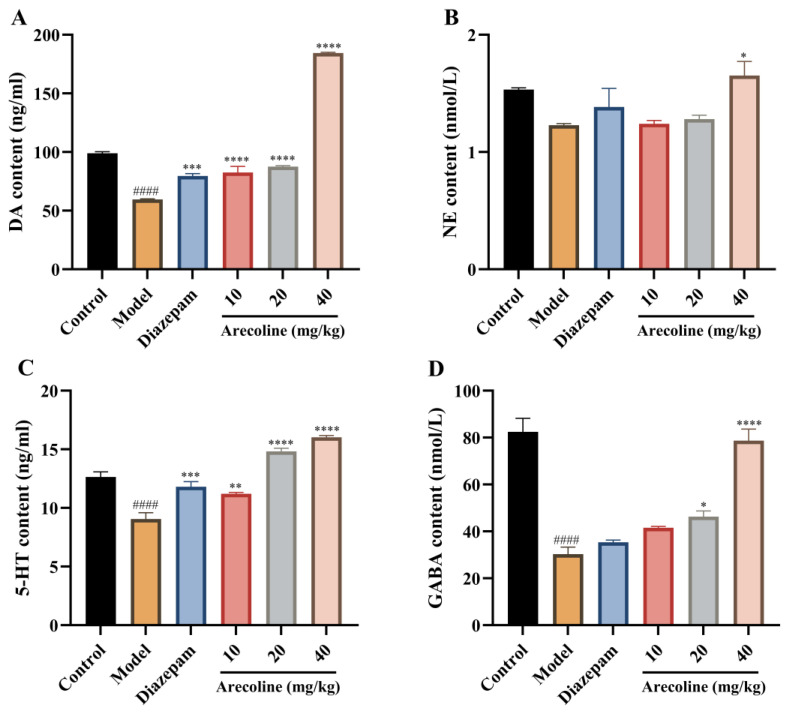
Effects of arecoline on hippocampal neurotransmitter levels in CUMS-induced mice. (**A**) DA content, (**B**) NE content, (**C**) 5-HT content, and (**D**) GABA content. Values represent the mean ± SEM (*n* = 12). #### *p* < 0.0001 vs. control; * *p* < 0.05, ** *p* < 0.01, *** *p* < 0.001, **** *p* < 0.0001 vs. model.

As illustrated in Figure 5A–D, CUMS caused a marked decline in hippocampal DA, 5-HT, and GABA contents (#### *p* < 0.0001) versus controls, whereas NE remained relatively unchanged. These alterations indicate neurotransmitter imbalance associated with chronic stress exposure. Administration of diazepam significantly restored DA and 5-HT levels (*** *p* < 0.001, ** *p* < 0.01) and partially enhanced GABA content. Treatment with arecoline (10, 20, 40 mg/kg) markedly reversed these neurochemical disturbances. In particular, the 10 and 20 mg/kg doses increased DA and 5-HT, while the 40 mg/kg group elevated all four neurotransmitters, including GABA (** *p* < 0.0001) and NE (*p* < 0.05). Together, these results confirm that arecoline alleviates CUMS-induced depletion of monoaminergic and inhibitory neurotransmitters, thereby contributing to its anxiolytic-like effects.

### 2.6. Effects of Arecoline on Oxidative Stress Markers in the Hippocampus of Mice Subjected to CUMS

**Figure 6:**
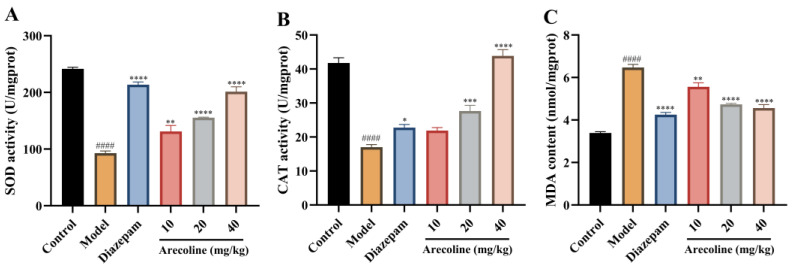
Effects of arecoline on oxidative stress markers in the hippocampus of CUMS-induced mice. (**A**) SOD activity; (**B**) CAT activity; (**C**) MDA content. Values represent the mean ± SEM (*n* = 12). #### *p* < 0.0001 vs. control; * *p* < 0.05, ** *p* < 0.01, *** *p* < 0.001, **** *p* < 0.0001 vs. model.

As illustrated in Figure 6A–C, CUMS markedly suppressed hippocampal SOD and CAT activities (#### *p* < 0.0001), accompanied by a pronounced elevation in MDA levels (#### *p* < 0.0001). Treatment with diazepam partially normalized these parameters by enhancing antioxidant enzyme activities and lowering lipid peroxidation. Administration of arecoline (10, 20, 40 mg/kg) similarly restored redox balance in the hippocampus. Across all doses, arecoline significantly increased SOD and CAT activities (** *p* < 0.01 to **** *p* < 0.0001) while reducing MDA accumulation (** *p* < 0.01 to **** *p* < 0.0001). The 40 mg/kg group showed the most evident recovery, suggesting that arecoline attenuates oxidative stress by reinforcing endogenous antioxidant capacity and limiting lipid peroxidation.

### 2.7. Effects of Arecoline on Inflammatory Cytokine Levels in the Hippocampus of Mice Subjected to CUMS

**Figure 7:**
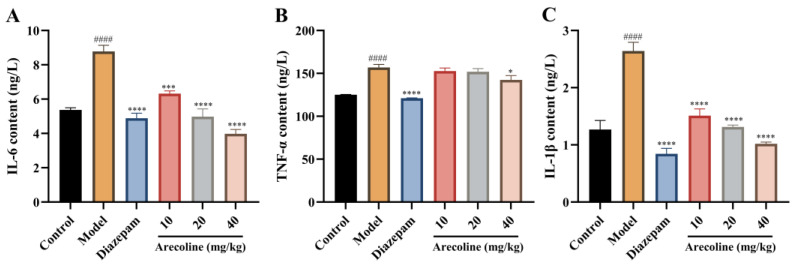
Effects of arecoline on hippocampal inflammatory cytokine levels in CUMS-induced mice. (**A**) IL-6 content; (**B**) TNF-α content; (**C**) IL-1β content. Values represent the mean ± SEM (*n* = 12). #### *p* < 0.0001 vs. control; * *p* < 0.05, *** *p* < 0.001, **** *p* < 0.0001 vs. model.

As presented in Figure 7A–C, exposure to CUMS resulted in a pronounced increase in hippocampal IL-6, TNF-α, and IL-1β levels (#### *p* < 0.0001) relative to the control group. Treatment with diazepam markedly reduced these cytokine elevations (**** *p* < 0.0001), demonstrating its known anti-inflammatory efficacy. Administration of arecoline (10, 20, 40 mg/kg) also significantly lowered hippocampal IL-6, TNF-α, and IL-1β concentrations, with reductions in IL-6 and IL-1β (*** *p* < 0.001 to **** *p* < 0.0001) and a gradual decrease in TNF-α (* *p* < 0.05 to **** *p* < 0.0001). The 40 mg/kg group exhibited the most evident normalization of cytokine levels. These results indicate that arecoline alleviates hippocampal inflammation from CUMS, confirming an anti-inflammatory pathway for its anxiety-reducing properties.

### 2.8. Effects of Arecoline on BDNF/TrkB/CREB Signaling in the Hippocampus of Mice Subjected to CUMS

**Figure 8:**
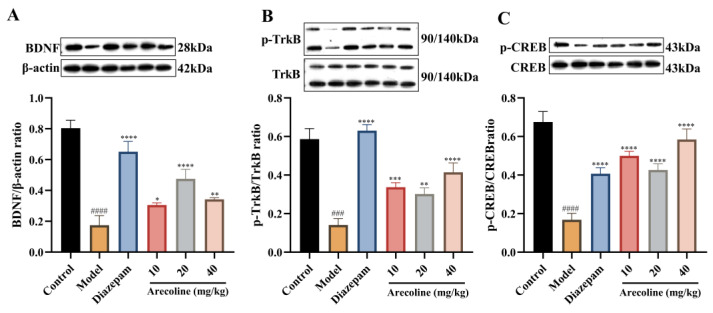
Effects of arecoline on the BDNF/TRKB/CREB signaling pathway in the hippocampus of CUMS-induced mice. (**A**) BDNF/β-actin ratio; (**B**) p-TrkB/TrkB ratio; (**C**) p-CREB/CREB ratio. For total protein expression, band intensities were normalized to β-actin. Phosphorylated protein levels were expressed as ratios to their corresponding total proteins. Values represent mean ± SEM (*n* = 3). ### *p* < 0.001, #### *p* < 0.0001 vs. control; * *p* < 0.05, ** *p* < 0.01, *** *p* < 0.001, **** *p* < 0.0001 vs. model.

As displayed in Figure 8A–C, exposure to CUMS markedly decreased hippocampal protein expression of BDNF, p-TrkB/TrkB, and p-CREB/CREB (#### *p* < 0.0001) compared with the control group, reflecting disrupted neurotrophic signaling under chronic stress conditions. Treatment with diazepam significantly restored these protein levels (**** *p* < 0.0001), consistent with its neuroprotective and anxiolytic actions. Likewise, arecoline administration (10, 20, 40 mg/kg) effectively counteracted the CUMS-induced downregulation of these signaling molecules. In particular, BDNF expression was elevated (* *p* < 0.05 to **** *p* < 0.0001), and both p-TrkB/TrkB and p-CREB/CREB ratios were markedly increased (*** *p* < 0.001 to **** *p* < 0.0001), with the most pronounced improvement observed in the 40 mg/kg group. These observations indicate that arecoline reactivates hippocampal BDNF/TrkB/CREB signaling, which may promote synaptic plasticity and ameliorate anxiety-like behavior in mice subjected to chronic stress.

### 2.9. Effects of Arecoline on NMDAR/CaMKII Signaling in the Hippocampus of Mice Subjected to CUMS

**Figure 9:**
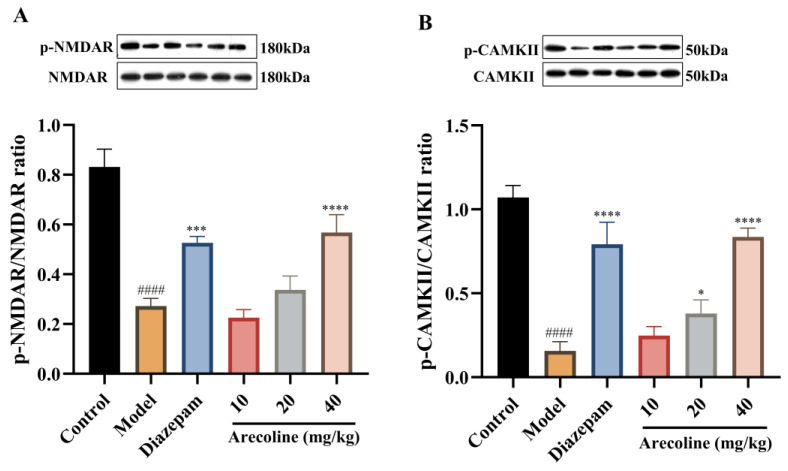
Effects of arecoline on the NMDAR/CaMKII signaling pathway in the hippocampus of CUMS-induced mice. (**A**) p-NMDAR/NMDAR ratio; (**B**) p-CaMKII/CaMKII ratio. Phosphorylated protein levels were quantified as ratios of phosphorylated to total protein. Values represent the mean ± SEM (*n* = 3). #### *p* < 0.0001 vs. control; * *p* < 0.05, *** *p* < 0.001, **** *p* < 0.0001 vs. model.

As displayed in Figure 9A,B, exposure to CUMS markedly reduced the phosphorylation levels of NMDAR and CaMKII compared with the control group (#### *p* < 0.0001), indicating suppressed synaptic activity and impaired excitatory signaling in the hippocampus. Treatment with diazepam significantly restored phosphorylation of both proteins (*** *p* < 0.001, **** *p* < 0.0001), confirming its neuroprotective effect. Administration of arecoline (10, 20, 40 mg/kg) also enhanced NMDAR and CaMKII phosphorylation, with the 40 mg/kg group exhibiting the most prominent improvement (* *p* < 0.05 to **** *p* < 0.0001). These findings indicate that arecoline reactivates hippocampal NMDAR/CaMKII signaling, which may restore synaptic responsiveness and support neuronal resilience under chronic stress conditions.

## 3. Discussion

In this study, we comprehensively evaluated the anxiolytic-like effects of arecoline in a CUMS mouse model through behavioral, biochemical, and molecular analyses. Treatment with arecoline markedly alleviated anxiety-like behaviors and reversed stress-induced neurobiological alterations. Specifically, arecoline restored behavioral performance in the OFT, LDB, and EPM tests, normalized hippocampal CORT levels, rebalanced key neurotransmitters (DA, NE, 5-HT, and GABA), enhanced antioxidant enzyme activity, reduced inflammatory cytokine expression, and upregulated neuroplasticity-related pathways (BDNF/TRKB/CREB and NMDAR/CaMKII). Collectively, these results demonstrate that arecoline exerts multifaceted neuroprotective and anxiolytic-like effects under chronic stress conditions, through coordinated regulation of neuroendocrine, neurochemical, oxidative, and inflammatory systems.

Behavioral assessments collectively indicated anxiolytic-like behavioral modulation by arecoline under CUMS conditions. In the OFT, CUMS-exposed mice exhibited markedly reduced central locomotion and exploratory activity, indicative of heightened anxiety and emotional inhibition. Similarly, in the LDB test, stressed mice spent less time in the illuminated chamber and made fewer transitions between compartments, reflecting increased avoidance behavior and reduced exploratory drive. These behavioral patterns align with previous studies showing that chronic stress diminishes exploratory motivation and promotes anxiety-related avoidance in rodents [26,27,28]. In the EPM, similar behavioral trends were observed. CUMS-exposed mice showed reduced open-arm exploration, a commonly reported feature of anxiety-like behavior [29]. Arecoline treatment increased the percentage of time spent in, and entries into, the open arms, suggesting a partial normalization of anxiety-related avoidance behavior. Together with the LDB and OFT findings, these results are consistent with anxiolytic-like effects of arecoline under CUMS conditions. These findings suggest that arecoline effectively counteracts stress-induced anxiety without inducing nonspecific motor impairment, thereby supporting its behavioral specificity. It should be noted that previous studies suggest that arecoline does not induce gross locomotor impairment in non-stressed animals [30]. As the present study did not include an arecoline-alone group, the behavioral effects observed here should be interpreted as context-dependent modulation of stress-induced behavioral alterations under CUMS conditions, rather than intrinsic psychostimulant effects of arecoline. Accordingly, the present findings do not allow conclusions regarding the intrinsic pharmacological effects of arecoline in non-stressed animals, but rather highlight its modulatory role in correcting stress-induced behavioral dysregulation.

Dysregulation of the HPA axis represents a key mechanism linking stress exposure to emotional disorders. Sustained stress leads to excessive systemic corticosterone release, which promotes oxidative stress, inflammation, and neuronal dysfunction [31,32,33]. In line with these reports, we observed elevated serum CORT levels in CUMS mice, confirming neuroendocrine hyperactivation. Arecoline treatment significantly lowered circulating CORT concentrations, indicating partial normalization of stress hormone output and restoration of systemic neuroendocrine homeostasis. As glucocorticoids influence neurotransmitter metabolism and synaptic excitability, this restoration may contribute to the behavioral recovery observed in arecoline-treated mice. Thus, arecoline appears to act initially by stabilizing systemic HPA axis activity, which forms the foundation for downstream molecular protection.

Neurotransmitter imbalance is another major feature of stress-related neuropsychiatric disorders [34]. Chronic stress disrupts serotonergic, dopaminergic, noradrenergic, and GABAergic signaling, leading to impaired emotional regulation and anxiety [35]. Consistent with this, CUMS exposure significantly altered hippocampal neurotransmitter levels, while arecoline restored 5-HT, DA, NE, and GABA toward normal values. Importantly, the anxiolytic efficacy of many established pharmacological agents has been closely linked to their ability to modulate central neurotransmitter systems. For example, benzodiazepines exert anxiolytic effects primarily by enhancing inhibitory GABAergic transmission through positive allosteric modulation of GABA_A receptors, thereby increasing inhibitory neurotransmission in the central nervous system [36]. In contrast, selective serotonin reuptake inhibitors increase serotonergic tone, which can secondarily influence dopaminergic and noradrenergic signaling and contribute to their therapeutic effects in anxiety disorders, consistent with previous reports indicating that serotonergic mechanisms represent a central substrate of anxiolytic action [37]. Together, these findings support the concept that anxiety-related behaviors arise from a disruption in the balance between excitatory and inhibitory neurotransmission, rather than dysfunction of a single neurotransmitter pathway, in line with contemporary models of anxiety pathophysiology [38]. Previous studies have shown that arecoline interacts with muscarinic and nicotinic acetylcholine receptors, influencing monoaminergic transmission and neural excitability [39,40]. Through functional interactions between acetylcholine receptors and GABAergic systems, cholinergic signaling has been shown to regulate GABA release and inhibitory tone, providing a plausible indirect mechanism for the GABAergic changes observed after arecoline treatment [41]. In this context, the concurrent restoration of hippocampal monoamines and GABA observed in the present study suggests that arecoline may alleviate anxiety-like behavior by rebalancing stress-disrupted neurochemical homeostasis through cholinergic regulation, rather than by targeting a single neurotransmitter system.

Oxidative stress is a well-recognized contributor to the neuropathology of chronic stress and anxiety [42]. Increased lipid peroxidation and reduced antioxidant enzyme activity are common features in the hippocampus of stressed rodents [43,44]. Our results support this pattern: CUMS elevated MDA levels while decreasing SOD and CAT activities. Arecoline administration restored antioxidant enzyme function and reduced lipid peroxidation under CUMS conditions, suggesting reinforcement of endogenous redox homeostasis. Because oxidative stress can initiate neuronal injury and activate inflammatory cascades, this antioxidative protection likely serves as a critical upstream event mitigating neuroinflammatory activation.

Neuroinflammation also plays a pivotal role in the development of anxiety-related pathology [45]. Elevated TNF-α, IL-6, and IL-1β levels observed in CUMS mice are consistent with previous reports showing that proinflammatory cytokines impair neuronal signaling and synaptic plasticity [46,47,48,49]. Under CUMS conditions, arecoline administration markedly suppressed these cytokines, confirming its anti-inflammatory potential in the central nervous system. Given that oxidative stress and inflammation form a self-reinforcing feedback loop, the simultaneous attenuation of both processes by arecoline suggests that its neuroprotective activity results from broad stabilization of the redox–immune interface.

Beyond biochemical modulation, chronic stress impairs neuroplasticity and synaptic signaling pathways critical for emotional adaptation [50]. Reductions in BDNF, TrkB, CREB, and related plasticity markers are strongly linked to stress-induced behavioral deficits [51,52]. Consistent with previous studies, CUMS markedly downregulated BDNF, TrkB, and CREB expression in the hippocampus, leading to synaptic dysfunction and anxiety-like behavior [53,54]. These observations align with established evidence that disruption of neurotrophic signaling is a core mechanism of stress-related neuropathology. In the present study, arecoline treatment reversed these alterations in CUMS-exposed mice by enhancing hippocampal BDNF expression and promoting phosphorylation of TrkB and CREB, indicating partial restoration of neurotrophic signaling capacity. Such reactivation of the BDNF/TrkB/CREB axis likely contributes to the recovery of emotional and cognitive function observed in arecoline-treated mice. In addition to neurotrophic modulation, restoration of excitatory synaptic signaling further supports the pro-plasticity effects of arecoline. Arecoline also upregulated phosphorylation of NMDAR and CaMKII, two critical regulators of glutamatergic synaptic transmission and long-term potentiation. Chronic stress typically suppresses NMDAR/CaMKII activation, impairing postsynaptic signaling and learning processes [55,56]. The restoration of these pathways in arecoline-treated mice suggests improved excitatory synaptic efficiency and potential cross-talk with BDNF/CREB signaling. Together, activation of the BDNF/TrkB/CREB and NMDAR/CaMKII pathways may act synergistically to reinforce synaptic plasticity and emotional stability following CUMS exposure, highlighting the integrated modulation of neurotrophic and glutamatergic signaling.

Although arecoline has been reported to show toxicological effects [57], no overt signs of discomfort or distress were observed in mice treated with arecoline (10–40 mg/kg) under CUMS conditions in the present study. These findings should be interpreted as pharmacological effects under chronic stress conditions rather than a comprehensive assessment of toxicity.

In summary, arecoline mitigates CUMS-induced anxiety through multi-level regulation of the systemic HPA axis, neurotransmitter balance, oxidative stress, inflammation, and neuroplasticity. By linking behavioral recovery with circulating corticosterone normalization and downstream molecular mechanisms, this study expands the pharmacological understanding of arecoline and provides mechanistic support for its traditional phytomedicinal use. Further research should focus on pharmacokinetics, long-term safety, and potential formulation strategies to facilitate its development as a neuroprotective phytochemical candidate.

## 4. Materials and Methods

### 4.1. Materials

Arecoline (CAS 63-75-2; ≥98% purity by HPLC; Cat. A14660) was obtained from Shanghai Jizhi Biochemical Technology Co., Ltd. (Shanghai, China). Diazepam tablets (Batch No. 220802) were supplied by Shandong Xinyi Pharmaceutical Co., Ltd. (Dezhou, China).

Assay kits for superoxide dismutase (SOD), malondialdehyde (MDA), and catalase (CAT) were purchased from Jianglai Biological Technology Co., Ltd. (Shanghai, China). ELISA kits for CORT, 5-HT, DA, NE, GABA, tumor necrosis factor-α (TNF-α), interleukin-1β (IL-1β), and interleukin-6 (IL-6) were obtained from Jiancheng Bioengineering Institute (Nanjing, China).

Primary antibodies against CREB, NMDAR, and CaMKII were purchased from Cell Signaling Technology (Boston, MA, USA), while those against BDNF and TrkB were supplied by Proteintech Group, Inc. (Wuhan, China). Secondary antibodies were purchased from Jackson ImmunoResearch Laboratories (West Grove, PA, USA).

### 4.2. Ethical Statement

The use of animals in this study was reviewed and approved by the Institutional Animal Care and Use Committee of Hunan Prima Pharmaceutical Research Center (approval number: HNSE2023(5)007, 25 May 2023). All procedures followed relevant national and institutional guidelines for laboratory animal welfare. Experimental mice were obtained from Beijing Weitong Lihua Laboratory Animal Technology Co., Ltd. (Beijing, China) (license No. SCXK (Yue) 2019-0063; certificate No. 44829700011576) and maintained under specific pathogen-free (SPF) conditions (SYXK (Xiang) 2020-0015).

### 4.3. Animals and Experimental Protocol for CUMS Induction

**Figure 10:**
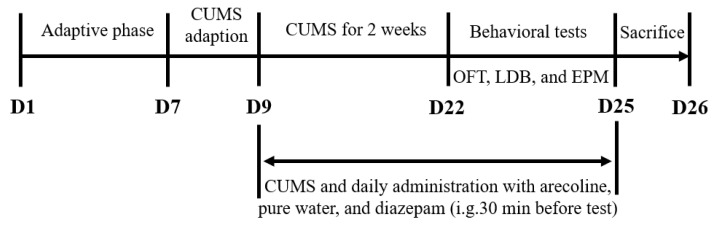
Experimental design and timeline of the CUMS mouse model and drug administration.

The overall experimental design and timeline of the CUMS protocol and drug administration are illustrated in Figure 10. After one week of acclimatization, 72 SPF male C57BL/6 mice (18.0–22.0 g) were randomly divided into six groups (*n* = 12):

(1) Control group (no stress, purified water);
(2) Model group (CUMS, purified water);
(3) Diazepam group (CUMS, diazepam 2.5 mg/kg, positive control);
(4) Arecoline low-dose group (CUMS, arecoline 10 mg/kg);
(5) Arecoline medium-dose group (CUMS, arecoline 20 mg/kg);
(6) Arecoline high-dose group (CUMS, arecoline 40 mg/kg).

During the 14-day modeling period, animals were exposed to a series of mild and varied stressors. The stressors included restraint, cage tilting at 45°, food or water deprivation, wet bedding, cold-water swimming at 4 °C for 10 min, strobe light exposure, and day–night reversal. Two to three different stressors were randomly applied each day, and no stressor was repeated on consecutive days to prevent adaptation. All mice were administered daily oral gavage (0.2 mL/10 g) throughout the experimental period.

Diazepam served as the positive control because of its well-documented anxiolytic efficacy and extensive use in rodent models of anxiety. As a classical benzodiazepine, diazepam exerts its anxiolytic effects primarily by enhancing GABAA receptor activity, and is commonly employed as a reference compound for evaluating the pharmacological potential of novel anxiolytic agents [58]. The arecoline dosages (10, 20, and 40 mg/kg) were chosen from our prior research, in which the same concentrations exerted significant neuroprotective and anti-fatigue effects in a sleep deprivation-induced mouse model [18] These doses were further confirmed in preliminary screening to be safe and well-tolerated, with no signs of toxicity or body-weight loss. Therefore, 10, 20, and 40 mg/kg were designated as low, medium, and high doses to evaluate dose-dependent anxiolytic efficacy in the present CUMS model.

### 4.4. Behavioral Tests

#### 4.4.1. Open Field Test (OFT)

The OFT provided an initial evaluation of general locomotor function and emotional exploration. Each mouse was placed in the center of a 50 cm × 50 cm × 40 cm square arena and permitted to roam freely for 5 min. Behavioral performance was recorded and analyzed using a computer-assisted video tracking system (model DigBehv, Shanghai Jiliang Software Technology Co., Ltd., Shanghai, China). Total distance, average speed, and activity time were quantified to reflect locomotor ability and anxiety-related behavior.

#### 4.4.2. Light–Dark Box (LDB) Test

To further evaluate anxiety tendencies, the LDB apparatus contained two connected chambers—a brightly illuminated compartment (27 cm × 18 cm × 27 cm, approximately 400 lux) and a dark compartment (18 cm × 18 cm × 27 cm, <10 lux)—linked by a 7 cm × 7 cm passage. Each animal started in the light side and was allowed to explore for 5 min. Time spent in the light compartment, percentage of time spent in the light compartment, and the number of transitions between the light and dark compartments were automatically recorded and analyzed using a computer-assisted video tracking system (model DigBehv).

#### 4.4.3. Elevated Plus Maze (EPM) Test

The EPM was employed as an additional behavioral assay to further evaluate anxiety-related behavior. The experimental apparatus consisted of a plus-shaped maze elevated 50 cm from the ground, featuring two uncovered arms (30 cm × 5 cm) and two walled arms (30 cm × 5 cm × 15 cm) extending from a central square platform (5 cm × 5 cm). Each mouse was placed at the center facing an open arm and given five minutes to explore freely. Behavioral performance was recorded and analyzed using a computer-assisted video tracking system specifically designed for the EPM paradigm (model JL BehvEPMG, Shanghai Jiliang Software Technology Co., Ltd., Shanghai, China). Percentage of open arm entries and percentage of time spent in open arms were recorded for subsequent analysis.

### 4.5. Sample Collection

After the behavioral tests, mice were anesthetized with 10% chloral hydrate. Blood samples were collected, allowed to clot at room temperature, and centrifuged to obtain serum. Serum samples were stored at −80 °C until analysis. The hippocampal tissues were collected and stored at −80 °C for subsequent analyses.

### 4.6. Biochemical Parameter Assays

Hippocampal antioxidant and oxidative stress markers, including SOD and CAT activities and MDA levels, were determined using commercial biochemical assay kits according to the manufacturers’ instructions. Hippocampal neurotransmitters (5-HT, DA, NE, and GABA) and pro-inflammatory cytokines (TNF-α, IL-1β, and IL-6) were quantified using commercial ELISA kits. Serum CORT levels were measured using an ELISA kit following the manufacturer’s protocol.

### 4.7. Western Blot Analysis

The Western blot procedure was executed as per the standard protocols, with a few tweaks [59]. The hippocampal samples were then blended in a lysis buffer enriched with phosphatase blockers to maintain protein phosphorylation integrity. Following a spin at 12,000 rpm for a solid 15 min at icy cold temperatures, we carefully extracted the resultant supernatant for further investigation.

Equal protein quantities (30 μg per lane) were separated via SDS-PAGE using an 8% resolving gel with a 5% stacking layer. The samples initially ran at 90 V for 20 min before ramping up to 120 V until clear band separation was achieved. Post-electrophoresis, proteins migrated to 0.45 μm PVDF membranes via a standard wet transfer system at 300 mA for 60 min.

To reduce nonspecific binding, the samples were pre-blocked for an hour using a 5% bovine serum albumin (BSA) solution in Tris-buffered saline (TBST) with 0.1% Tween-20, at ambient temperature. Subsequently, they underwent a 24-h incubation period in the refrigerator at 4 degrees Celsius, utilizing the same 5% BSA–TBST mixture to dilute the primary antibodies. Post-washing in TBST three times, we applied HRP-linked secondary antibodies at a 1:10,000 ratio for an hour at room temp. We spotted the immunoreactive bands with ECL reagents and captured their images via a chemiluminescence reader, tweaking the exposure time from 10 s to 5 min to match the intensity of the signals.

Protein expression levels were quantified using Image-Pro Plus (IPP 6.0; Media Cybernetics, Rockville, MD, USA). For total protein expression (BDNF), band intensities were normalized to β-actin as the loading control. For phosphorylated proteins, the ratios of phosphorylated to total protein (p-TrkB/TrkB, p-CREB/CREB, p-NMDAR/NMDAR, and p-CaMKII/CaMKII) were calculated.

### 4.8. Statistical Analysis

Results are presented as mean ± standard error of the mean (SEM). All statistical analyses were performed using GraphPad Prism version 8.01 (GraphPad Software, San Diego, CA, USA). Group differences were evaluated using one-way analysis of variance (ANOVA). When the omnibus ANOVA indicated a statistically significant effect (*p* < 0.05), Dunnett’s multiple comparisons test was applied for planned comparisons, with the CUMS model group used as the reference, to compare the control group and each treatment group against the model group. A two-tailed *p* < 0.05 was considered statistically significant.

## 5. Conclusions

In conclusion, our study demonstrated that arecoline effectively alleviated anxiety-like behaviors and neuronal damage in CUMS-induced mice. Arecoline treatment restored HPA axis stability, normalized neurotransmitter profiles (5-HT, DA, NE, GABA), and improved antioxidant capacity while suppressing oxidative stress and inflammatory responses. At the molecular level, arecoline activated the BDNF/TrkB/CREB and NMDAR/CaMKII signaling pathways, thereby promoting hippocampal synaptic plasticity and maintaining neuronal homeostasis. These integrated effects reveal the multi-target regulatory role of arecoline in mitigating CUMS-induced neurobehavioral impairments. Collectively, our findings provide mechanistic insight into the neuroprotective potential of arecoline and support its development as a natural therapeutic candidate for anxiety disorders associated with chronic stress.

## Data Availability

The original contributions presented in the study are included in the article; further inquiries can be directed to the corresponding authors.

## Abbreviations

The following abbreviations are used in this manuscript:

BDNF	Brain-Derived Neurotrophic Factor
CaMKII	Calcium/Calmodulin-Dependent Protein Kinase II
CAT	Catalase
CORT	Corticosterone
CREB	cAMP Response Element-Binding Protein
CUMS	Chronic Unpredictable Mild Stress
DA	Dopamine
ELISA	Enzyme-Linked Immunosorbent Assay
GABA	Gamma-Aminobutyric Acid
GAPDH	Glyceraldehyde-3-Phosphate Dehydrogenase
HPA	Hypothalamic–Pituitary–Adrenal (Axis)
IL-1β	Interleukin-1β
IL-6	Interleukin-6
LDB	Light–Dark Box
MDA	Malondialdehyde
NE	Norepinephrine
NMDAR	N-Methyl-D-Aspartate Receptor
OFT	Open Field Test
ROS	Reactive Oxygen Species
SOD	Superoxide Dismutase
SPF	Specific Pathogen-Free
TNF-α	Tumor Necrosis Factor-Alpha
TrkB	Tropomyosin Receptor Kinase B
5-HT	5-Hydroxytryptamine (Serotonin)
